# Acute mechanical, physiological and perceptual responses in older men to traditional-set or different cluster-set configuration resistance training protocols

**DOI:** 10.1007/s00421-020-04453-y

**Published:** 2020-08-10

**Authors:** Antonio Dello Iacono, Domenico Martone, Lawrence Hayes

**Affiliations:** 1 University of the West of Scotland (School of Health and Life Sciences), Hamilton, UK; 2 Universita’ Degli Studi di Napoli (Dipartimento di Science Motorie e Benessere), Naples, Italy

**Keywords:** Elderly, Health, Resistance training, Strength, Power

## Abstract

**Purpose:**

The aims of this study were to compare mechanical outputs (i.e. power and impulse), physiological (i.e. heart rate) and perceptual (i.e. effort and fatigue) responses in older men to traditional-set or different cluster-set configuration resistance training protocols.

**Methods:**

In a randomized cross-over design, 20 healthy old men (aged 67.2 ± 2.1 years) completed four resistance training sessions using the back squat exercise loaded with optimal power loads. Training configurations were: traditional (TRA), three sets of six repetitions with 120-s rest between each set; Cluster-set 1 (CLU1), 24 single-repetition clusters with 10 s of rest after every cluster; Cluster-set 2 (CLU2), 12 double-repetition clusters with 20-s rest after every cluster; and Cluster-set 4 (CLU4), 6 quadruple-repetition clusters with 40-s rest after every cluster.

**Results:**

Cluster-set configurations resulted in greater power outputs compared to traditional-set configuration (range 2.6–9.2%, all *p* $\le$ 0.07 for main effect and protocol $\times$ set interactions). CLU1 and CLU2 induced higher heart rate (range 7.1–10.5%, all *p* < 0.001 for main effect and protocol $\times$ set interactions), lower rating of perceived exertion (range − 1.3 to − 3.2 AU, all *p* $\le$ 0.006 for pairwise comparisons) and lower ratings of fatigue (range − 0.15 to − 4 AU, all *p* $\le$ 0.012 for pairwise comparisons) compared to TRA and CLU4. Finally, an absolute preference for CLU2 was reported.

**Conclusions:**

Findings presented here support the prescription of CLU2 as an optimal resistance training configuration for trained older men using the back squat.

## Introduction

Aging is a complex and multidimensional process characterized by a variety of biological changes, degenerative in nature, which contribute to impaired physiological processes. As such, a general decline in function with increased likelihood of adverse health outcomes ensues (Lally and Crome 2007). Even with healthy aging, functional capacity can decline as much as 40% between 60 and 90 years of age (Rikli and Jones 2013), commonly arising from factors such as skeletal muscle atrophy and reduced muscular strength. Low muscular strength causes functional challenges due to the reduced capability to produce forces necessary to accomplish activities of daily living. Importantly, the ability to exert high levels of force at high velocity (i.e. muscle power) decreases with advancing age faster than the muscle mass loss and muscular strength reduction (Metter et al. 1997; Skelton et al. 1994), due to a selective loss of Type II fibers in old age. These age-related impairments in skeletal muscle morphology, strength and more meaningfully, power capabilities, are part of the causal pathway for secondary adverse outcomes such as frailty, reduced mobility (Bischoff-Ferrari et al. 2015; Schaap et al. 2018), longer hospitalisation (Cawthon et al. 2017) and specific comorbidities including poorer bone health, osteoporosis (Bischoff-Ferrari et al. 2015; Schaap et al. 2018), obesity and type 2 diabetes (van Sloten et al. 2011). Moreover, a decline in muscle function is strongly associated with future physical disability and mortality from middle-age into later life (De Buyser et al. 2016).

Given the undesirable consequences of aging on the musculoskeletal system, strategies for preserving muscle function, muscular strength and muscle power are of great importance for the health and wellbeing of older adults. One common strategy to attenuate the effects of aging on neuromuscular function and functional capacity (Borde et al. 2015; Cadore et al. 2014; Peterson et al. 2010) is resistance training. Resistance training programs induce beneficial adaptations in both healthy older adults and those with chronic conditions if performed regularly (e.g., 2–3 days per week) in a periodized manner, with sufficient volume (e.g., 2–3 sets of 8–12 repetitions per exercise) and at an adequate intensity (e.g., 70–85% of 1 repetition maximum [1RM]) (Fragala et al. 2019; Medicine 2009; Pescatello et al. 2014). However, whereas these broad guidelines are commonly recommended to pursue muscle hypertrophy effects and general strength development (Fragala et al. 2019; Medicine 2009; Pescatello et al. 2014), approaches more oriented towards power enhancements could optimize the training responses underpinning greater functional improvements in older people. In support of this rationale, previous studies (Ramírez-Campillo et al. 2014, 2017; Ramirez-Campillo et al. 2016) have consistently reported power training protocols to induce greater improvements in functional performance outcomes and quality of life compared to traditional resistance training protocols in older people. In view of this accumulating evidence, investigating training protocols designed to optimize power output by manipulating resistance training variables seems prudent. One viable strategy to optimize the individual power responses to training is by using optimal power loads (OPL), accurately calculated for a specific resistance exercise and more importantly tailored to individual mechanical profiles (Loturco et al. 2015). Earlier studies conducted in athletic populations have reported greater power responses following resistance training regimens implementing OPL as opposed to protocols designed using absolute percentages of 1RM as reference (Dello Iacono et al. 2018; Dello Iacono and Seitz 2018; Oliver et al. 2016a). Another potential method to maximize power outputs in resistance training is through cluster-set configurations, which include short rest periods between repetitions within a given set. Cluster-set configurations can be designed by redistributing the repetitions within a given set into small clusters (e.g., 2–6 clusters of 2–4 of repetitions) separated by brief rest periods (e.g., 10–60 s). Cluster-set configurations facilitate greater force output, velocity and consequently power at a given load compared to traditional configuration (absent of intra-set or inter-repetition rest) in athletes (Tufano et al. 2016, 2017) and older adults alike (Ramirez-Campillo et al. 2018). Interestingly, cluster-sets also allow greater power output when compared to matched protocols loaded with the same OPL but designed as traditional-set configurations (Dello Iacono et al. 2019). The cumulative beneficial effects of OPL and cluster-set configurations on power output likely stem from psychophysiological (González-Hernández et al. 2020) and metabolic (Gorostiaga et al. 2014, 2010) mechanisms resulting in lower perceived effort and reduced acute muscular fatigue (Tufano et al. 2016, 2017). Furthermore, the rest interval between consecutive clusters significantly affects cardiovascular load (Fleck 1988, 2003; Kraemer et al. 1987), with very short rest periods (i.e. < 20 s) leading to higher heart rate (HR) responses. Therefore, it is worth identifying optimal set configurations (i.e. number of repetitions per cluster and associated inter-cluster rest duration) to induce beneficial cardiovascular responses alongside the known advantageous effects on acute muscular performance. In particular, the combination of cluster-sets and OPL may represent a viable method for optimizing power output and physiological responses in older adults performing resistance training. More importantly, optimization of power output could consequently lead to increased functional capacity and performance of activities of daily living.

The aims of this study were twofold: first, to compare mechanical responses to traditional-set or different cluster-set configuration resistance training protocols using OPL in older people. Second, assuming cluster-set configuration would optimize mechanical performance, we aimed to examine the underlying mechanisms by investigating physiological (i.e. HR) and perceptual responses (i.e. effort and fatigue). In view of the above, we hypothesize that resistance training protocols using OPL together with the cluster-set configurations would result in: (1) greater power outputs, (2) greater physiological responses (i.e. HR) and (3) lower perceived effort and fatigue, compared to a protocol configured in a traditional manner. To these ends, healthy old men completed four resistance training protocols, designed as traditional-set or cluster-set configurations, using the back squat exercise loaded with OPL.

## Materials and methods

### Subjects

Sample size was estimated using a priori power analysis in the G ∗ Power software (Heinrich-Heine-Universitat Dusseldorf, Germany). A one-way analysis of variance (ANOVA) with an *α* = 0.05, *β* = 0.8 and *large* effect size (ES = 0.86) (Dello Iacono et al. 2019) between traditional and cluster-set configured resistance training protocols loaded with OPL gave a statistical power of 82.4% and an estimated sample size of twenty subjects.

Twenty active healthy old men (aged 67.2 ± 2.1 years, with a height of 172.3 ± 5.8 cm, a body mass of 84.2 ± 9.3 kg, body mass index of 28.4 ± 2.1 kg/m^2^ and maximal oxygen uptake of 32.3 ± 2.7 mL/min/kg) were recruited via advertisement flyers distributed in local gym facilities. Medical screening was performed at their local general practice before commencement of the study. Exclusion criteria were symptoms or history of cardiovascular disease, hyperglycemia, diagnosed hypertension, any articular or musculoskeletal tissue injury of the trunk, lower back or lower limbs during the last 6 months. Subjects had a minmum of 2 years’ resistance training experience (range 2–4 years)s and at least 1 year of experience with the back squat exercise (range 1–3 years). They reported to train regularly, combining whole body resistance training, core training and aerobic conditioning, two to four times per week for approximately 90 min per session. Written informed consent was obtained following an oral explanation of the purpose, benefits and potential risks of the study. Procedures were conducted in accordance with the Declaration of Helsinki and approved by the Institution’s Ethics Committee of the University of the West of Scotland.

### Design

**Fig. 1:**
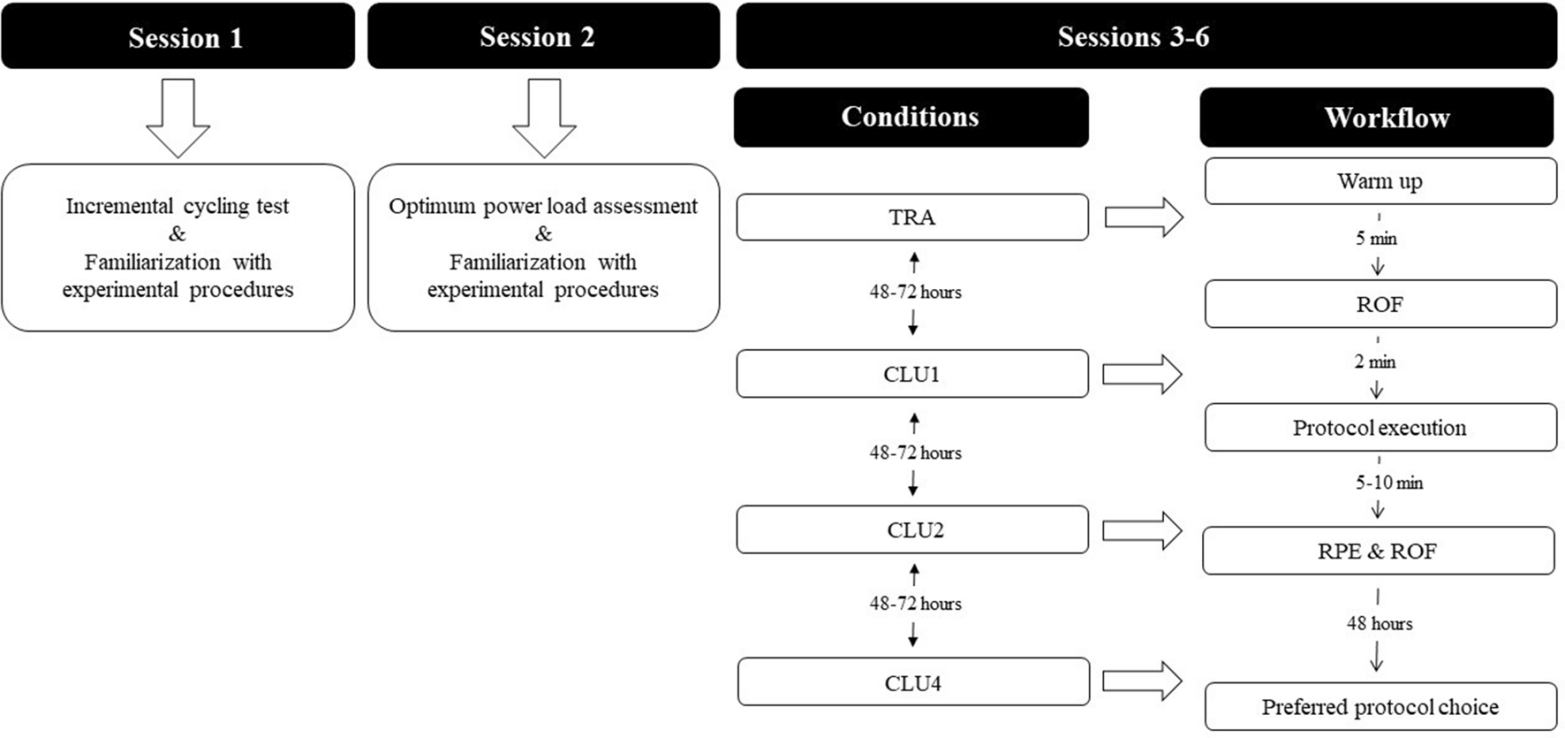
Schematic representation of the study design. *TRA* traditional-set configuration, *CLU1* single-repetition cluster configuration, *CLU2* double-repetition cluster configuration, *CLU4* quadruple-repetition cluster configuration, *RPE* rate of perceived effort, *ROF* rate of fatigue

To test our hypotheses, we used a randomized cross-over study design and compared the mechanical, physiological and perceptual responses to four resistance training protocols employing the same strength exercise (back squat) loaded with the same intensity (individual OPL), but designed with different set configurations (traditional or three different cluster-set protocols). Subjects completed two familiarization and four experimental sessions each including a standardized warm up, either a traditional or cluster-set protocols (Fig. 1 for the study layout). The order in which the protocols were completed was counter-balanced and determined by block randomization (www.random.org). Sessions were administered in the same gym facility, at the same time of the day (15:00–18:00 h), ambient temperature (21.7 ± 0.5 °C)s and relative humidity (60 ± 2%). Subjects were asked to maintain their normal diet throughout the study duration and to refrain from completing any strenuous physical activities and from consuming caffeine, alcohol, or any ergogenic substance 2 days prior and on the day of experimental sessions, respectively. All subjects performed the four experimental trials within 3 weeks and with 72–96 h between each session.

#### Maximal heart rate and optimum power load assessment

Two weeks prior to the study, subjects completed two testing sessions and were familiarized with experimental procedures. In the first familiarization session, after a standardized warm up protocol consisting of 4 min of treadmill walking (5 km/h) and 4 min of jogging (7 km/h) followed by 2 min of passive recovery and finally 2 min of dynamic mobilization exercises, subjects completed an incremental cycling test to exhaustion following a protocol previously described (Andersen et al. 2014). HR (bmp) was measured (Polar Team 2 System, Polar Electro Oy, Kempele, Finland) continuously throughout the exercise protocol and the individual maximal heart rate (HR_max_) was determined as the highest value measured within a 15-s period during the testing session. In the second familiarization session, the OPL in the back squat was determined for each subject. First, subjects performed the same standardized warm up described above. Then, back squat warm up sets with progressively heavier loads were performed. For the back squat execution, subjects were asked to keep the barbell constantly pressed against the shoulders blade, to push against the ground as hard and fast as possible during the upward movement. To mitigate variation in kinematic and kinetic patterns, back squat depth was standardized using an adjustable rod placed on a tripod. Moreover, the duration of the eccentric phase was preset at 3 s and dictated by a metronome to avoid confounding effects induced by inter-individual differences in pacing strategies. Subjects squatted down until touching the rod with their glutes before completing the upward movement of the back squat exercise. The OPL was determined following the protocol described by Loturco et al. (2015) on a Smith machine (Technogym Equipment, Italy). Specifically, the first absolute load used for the OPL assessment corresponded to an unloaded 20 kg barbell. Then, successive back squat trials with increasing loads (i.e., additional ~ 10% of body mass each trial) were performed until a decrease in the mean propulsive power (MPP) was observed. MPP only refers to the upward portion of the back squat during which barbell acceleration is greater than gravity (i.e., 9.81 m s^−2^). MPP has been previously reported as a preferable mechanical measure among power outputs in resistance training exercises as it limits biased underestimation of an individual’s power capabilities when lifting light or moderate loads (Sanchez-Medina et al. 2010). The OPL was identified as the back squat load with the highest MPP measured during trials. The same OPL was used in the four experimental sessions. MPP was determined using a validated (Vivancos et al. 2014) linear encoder (Chronojump, Barcelona, Spain) sampling at 1000 Hz, fixed to the bar of the Smith machine and computed using the software provided by the manufacturer in conjunction with the device.

### Experimental protocols

**Fig. 2:**
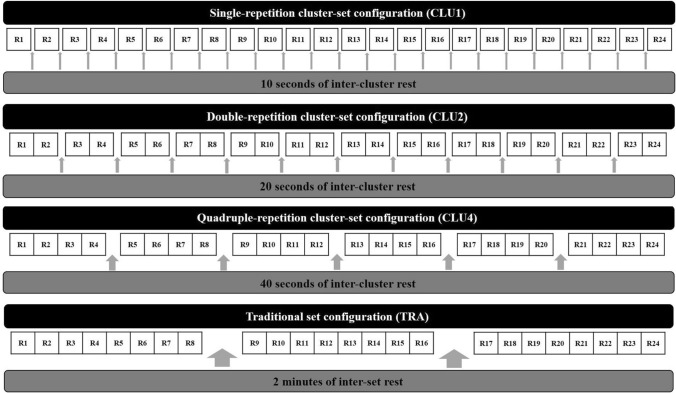
Traditional (TRA) and cluster-set configurations (CLU1, CLU2, and CLU4) investigated in the present study. *R* repetition

The resistance training session protocols consisted of free barbell back squats loaded with OPL and designed with the following sets, repetitions and rest configurations (Fig. 2):

- Traditional (TRA): three sets of eight repetitions (3 × 8) with two minutes of rest between each set.
- Cluster-set 1 (CLU1): 24 single-repetition clusters (24 × 1) with ten seconds of rest after every cluster.
- Cluster-set 2 (CLU2): 12 double-repetition clusters (12 × 2) with twenty seconds rest after every cluster.
- Cluster-set 4 (CLU4): six quadruple-repetition clusters (6 × 4) with forty seconds rest after every cluster.

With this approach, all protocols included the same total rest time during the training session. During experimental sessions, subjects completed the same standardized warm up used before the OPL assessments and followed the same instructions (i.e. position, range of motion and eccentric phase cadence) to execute the back squat exercise with a correct technique in a consistent manner. A researcher and two coaches supervised all sessions, providing verbal encouragement and instructing the subjects to focus on moving the bar as fast and as forcefully as possible by promoting an external focus of attention to elicit the greatest mechanical outputs (Dello Iacono et al. 2019). The duration of the protocols, including the rest intervals and duration of the sets, was about 6 minutes for all conditions.

### Physiological responses

HR was recorded at 1-s intervals using a heart rate monitor and short-range telemetry during each session. The HR monitor also ensured subjects started the experimental sessions with the same HR across conditions after completion of the standardized warm up. Data were collected for the duration of the experimental sessions, then pooled to determine average (%) HR responses expressed relatively to the individual HR_max_ for each protocol.

### Mechanical outputs

Mechanical outputs were collected with the linear encoder and software described above. Specifically, two measures were calculated; (1) the relative MPP (W $\bullet$ kg^−1^) during the propulsive upward portion of the back squat; and (2) the relative vertical impulse calculated from force–time curves as the force multiplied by time, from each repetition, expressed relative to body mass (kN/kg). Single repetition data for each measure was pooled to determine the mean for each protocol and used for statistical analysis.

### Perceptual responses

During familiarizations sessions, subjects were instructed how to use two different single-item scales to rate their subjective perceptions of fatigue and effort. Fatigue was measured via the 11-point rating-of-fatigue scale (ROF) (Micklewright et al. 2017). The question “How fatigued are you?” was presented at the top of the scale. The scale ranges from 0 (‘not fatigued at all’) to 10 (‘total fatigue and exhaustion–nothing left’). Subjects rated how fatigued they were before and within ten minutes of finishing each experimental protocol. Perceived effort was measured via an 11-point rating of perceived exertion scale (RPE) developed by Steele et al. (2016) as the instructions and anchors were sought to be best suited for the purpose of this study. The question “How much effort did you exert?” was presented at the top of the scale which ranged from 0 (‘no effort’) to 10 (‘maximal effort’). Subjects rated their effort within 5 min of completing each experimental protocol. Finally, 2 days after study completion, subjects were required to respond to a multiple-choice (single answer) online survey containing only one request: “Please select your favourite training protocol”. The order choices were randomized with five options presented horizontally: “traditional”, “cluster-1”, “cluster-2”, “cluster-4” and “none”.

### Statistical analysis

Data are presented as means ± standard deviation (SD) and confidence interval (95% CI). Figures are presented as grouped dot plots, as recommended by Drummond and Vowler (2012). Normality of the absolute data was investigated using the Shapiro–Wilk test, and skewness and kurtosis values smaller than two served as indication of normality (Leech and Onwuegbuzie 2002). Reliability of the baseline HR responses, MPP and impulse outputs produced in the first repetition across the four experimental protocols was assessed by calculating the Intra-class Correlation Coefficient (ICC_3,1_). Values, less than 0.5, between 0.5 and 0.75, between 0.75 and 0.9, and greater than 0.9 were interpreted as indicative of poor, moderate, good and excellent reliability, respectively. To complement the reliability analysis, a one-way Analysis of Variance (ANOVA) was used to compare the MPP and impulse outputs of the first repetition between the four protocols. HR responses at baseline and at the completion of each set were compared between the four protocols using a 4 (protocol: TRA, CLU4, CLU2, CLU1) × 4 (time point: baseline, set 1, set 2, set 3) repeated measures ANOVA. Comparisons between the four protocols on MPP and impulse outputs were tested using a 4 (protocol: TRA, CLU4, CLU2, CLU1) × 3 (set: set 1, set 2, set 3) repeated measures ANOVA. For this purpose, average MPP and impulse outputs were calculated separately for each set completed during each protocol. To test for differences between protocols on RPE, a one-way non-parametric ANOVA (Kruskal–Wallis *H* test) was used due to the violation of the normality. ROF was analyzed by comparing the difference between pre- and post-training protocol. The post-protocol values of each participant were subtracted from the baseline values within each condition. Then, these differences were compared using a one-way non-parametric ANOVA (Kruskal–Wallis *H* test) to examine differences between conditions whilst accounting for baseline differences. Significance was set at *p* < 0.05. 95% confidence intervals (CI) are reported alongside *p* values to allow for a better qualitative interpretation of the data (Cumming 2014; Dragicevic 2016). If the assumption of sphericity was violated, as indicated by Mauchly’s test, we employed a Greenhouse–Geiser correction. If significant main and interaction effects were identified, then post hoc pairwise comparisons were conducted using the Holm–Bonferroni correction. Finally, we inspected the linear relationship between HR responses (%HR_max_) measured at the end of the first set (i.e. at completion of the 8th repetition during each protocol) and the duration(s) of the same set across the four protocols (i.e. time interval calculated between the 1st and 8th repetitions) using two approaches. First, we plotted the individual data points ($x$ = duration, $y$ = HR) and visually confirmed a clear non-linear pattern between the dependent and explanatory variables. Second, we analyzed the relationship using a centered second order polynomial regression as follows:

$${{y}_{i}= \beta }_{0}+ {\beta }_{1}{x}_{i}+ {\beta }_{2}{{x}_{i}}^{2}+ \varepsilon ,$$

where ${y}_{i}$ denotes HR response of subject *i* at a given duration ${x}_{i}$; *β*_0_ is the coefficient of $y$ when ${x}_{i}$ = 0 and the duration of set 1 is equal to the average duration between protocols TRA and CLU4; *β*_1–2_ are the coefficients of the independent variable ${x}_{i}$ when ${x}_{i}$ > 0. Assumption of homoscedasticity was confirmed by visually inspecting the scatterplot of fitted values and residuals of the fitting model, from which no obvious pattern was identified. Assumption of normality was investigated using the D’Agostino-Pearson test. All statistical analyses were conducted using GraphPad Prism version 6.07 (GraphPad Software, La Jolla, California, USA).

## Results

ICCs for baseline HR, and first repetition MPP and first repetition impulse across the four protocols were 0.82 (95% CI 0.67, 0.92), 0.96 (95% CI 0.92, 0.98) and 0.76 (95% CI 0.72, 0.78), respectively. These results demonstrate good to excellent between-protocol reliability. No differences were found between experimental protocols for baseline HR responses (all *p* $\ge$ 0.91), MPP (all *p* $\ge$ 0.21) and impulse (all *p* $\ge$ 0.08) of the first repetition (Table 1 for descriptive statistics and 95% CI).

**Table 1:** Descriptive (mean ± SD) and inferential (95% CI) statistics of all variables, for all conditions

Variables	Condition			
	TRA	CLU4	CLU2	CLU1
***Mechanical outputs***				
*Mean propulsive power (W∙kg*^*−1*^*)*				
1st repetition	7.6 ± 0.21 (7.49, 7.69)	7.61 ± 0.2 (7.52, 7.7)	7.62 ± 0.2 (7.53, 7.72)	7.61 ± 0.19 (7.53, 7.7)
Set 1	6.79 ± 0.14 (6.73, 6.86)	6.96 ± 0.13^a^ (6.9, 7.02)	7.06 ± 0.2^a,f^ (6.96, 7.16)	7.28 ± 0.52^a,b,c^ (7.04, 7.52)
Set 2	6.64 ± 0.13 (6.58, 6.7)	6.76 ± 0.19^a^ (6.67, 7.84)	6.97 ± 0.2^a,b^ (6.87, 7.06)	7.25 ± 0.28^a,b,c^ (7.12, 7.39)
Set 3	6.51 ± 0.19 (6.42, 6.6)	6.76 ± 0.18 ^a^ (6.68, 7.84)	6.96 ± 0.21^a,b^ (6.87, 7.06)	7.23 ± 0.35^a,b,c^ (7.07, 7.39)
*Impulse (kN∙kg*^*−1*^*)*				
1st repetition	4.6 ± 0.14 (4.53, 4.67)	4.6 ± 0.16 (4.53, 4.68)	4.62 ± 0.15 (4.55, 4.69)	4.62 ± 0.17 (4.54, 4.7)
Set 1	4.7 ± 0.12 (4.65, 4.76)	4.74 ± 0.2 (4.65, 4.84)	4.72 ± 0.21 (4.62, 4.81)	4.75 ± 0.14 (4.69, 4.82)
Set 2	4.78 ± 0.14 (4.72, 4.85)	4.78 ± 0.18 (4.7, 4.87)	4.76 ± 0.18 (4.67, 4.84)	4.74 ± 0.11 (4.69, 4.8)
Set 3	4.9 ± 0.14^d,e^ (4.83, 4.97)	4.88 ± 0.14^d,e^ (4.8, 4.96)	4.81 ± 0.18 (4.73, 4.89)	4.8 ± 0.12 (4.75, 4.86)
***Physiological responses***				
*Heart rate (%HR*_*max*_*)*				
Baseline	42.1 ± 1.1 (41.6, 42.6)	42 ± 1 (41.5, 42.5)	42.2 ± 1 (41.7, 42.7)	42.2 ± 1 (41.7, 42.7)
Set 1	52 ± 1.5 (51.2, 52.6)	52.2 ± 1.7 (51.4, 53)	60.6 ± 1.7^a,b^ (59.8, 61.4)	60.9 ± 1.8^a,b^ (60, 61.7)
Set 2	55.7 ± 2.6 (54.5, 56.9)	56.3 ± 2.3 (55.5, 57.6)	64.2 ± 1.6^a,b^ (63.4, 64.9)	64.8 ± 1.7^a,b^ (64, 65.6)
Set 3	57.6 ± 2.5 (56.5, 58.8)	58.1 ± 2.5 (57, 59.3)	65.3 ± 1.8^a,b^ (64.4, 66.1)	66.2 ± 1.4^a,b^ (65.6, 66.8)
***Subjective responses***				
Rate of perceived exertion (AU)	6.7 ± 0.8 (6.4, 7.1)	4.1 ± 0.9^a,b^ (3.7, 4,4)	4.5 ± 0.7^a,b^ (4.2, 4.8)	6.5 ± 0.8 (6.1, 6.8)
Rate of fatigue (AU)	5.5 ± 1 (5.1, 5.9)	2.4 ± 1^a,b^ (1.9, 2.8)	2.6 ± 0.6^a,b^ (2.3, 2.9)	3.2 ± 0.6^a^ (2.9, 3.5)
Training preference	–	3	15	2

*CI* confidence intervals, *W* watts, *kg* kilograms, *k* kilo, *N* newton, *HR*_*max*_ maximal heart rate, *AU* arbitrary unit, *TRA* traditional, *CLU1* cluster-1, *CLU2* cluster-2, *CLU4* cluster-4

^a^Statistically (*p* < 0.01) different from TRA

^b^Statistically (*p* < 0.01) different from CLU4

^c^Statistically (*p* < 0.01) different from CLU2

^d^Statistically (*p* < 0.01) different from CLU1

^e^Statistically (*p* < 0.01) different from CLU2

^f^Statistically (*p* < 0.05) different from CLU4

**Fig. 3:**
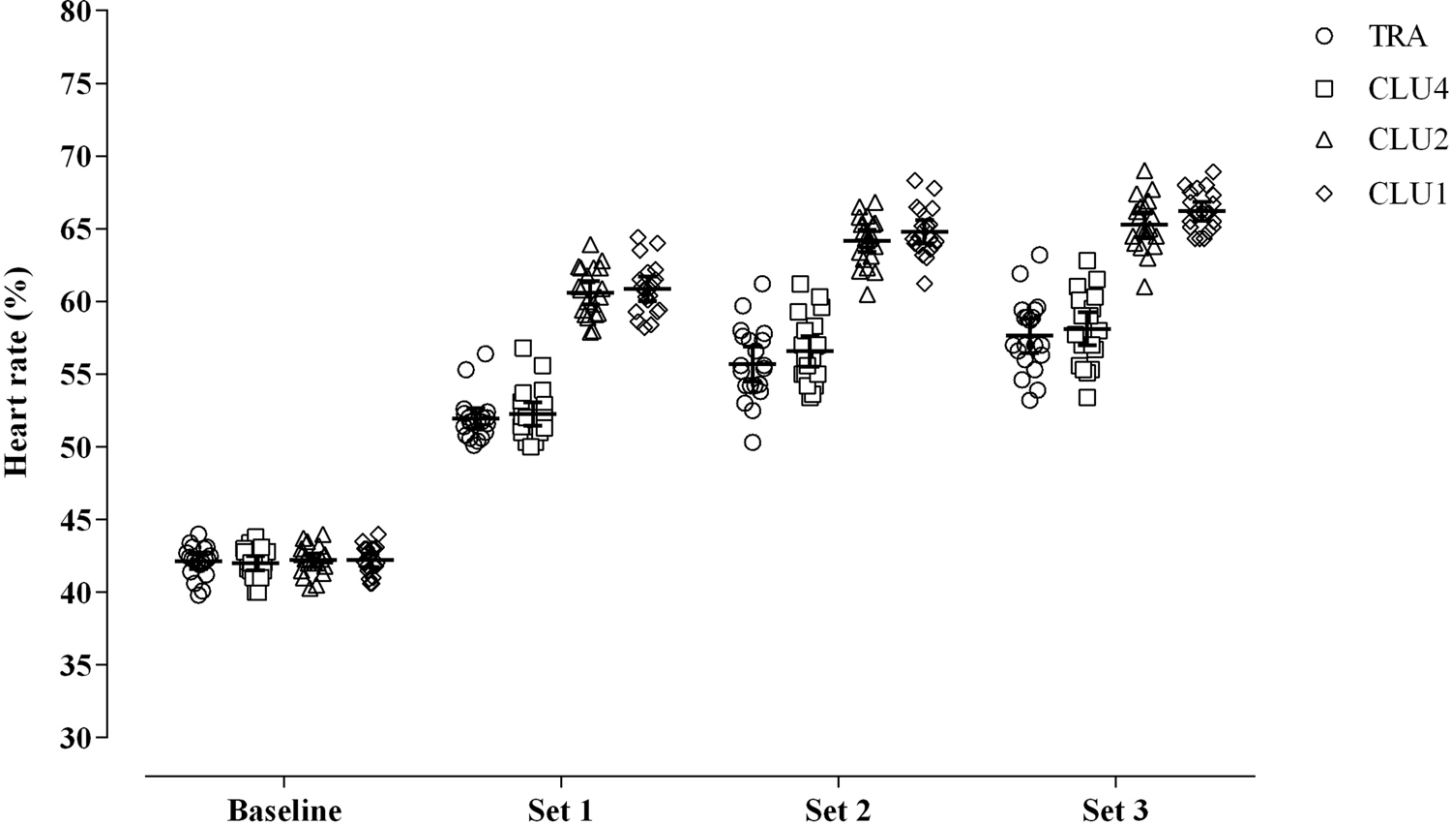
Comparison of the heart rate responses between the four different set configurations at baseline and across sets. TRA traditional-set configuration, *CLU1* single-repetition cluster configuration, *CLU2* double-repetition cluster configuration, *CLU4* quadruple-repetition cluster configuration

A main effect for protocol (*F*_(3, 76)_ = 181.5, *p* < 0.001) and interaction (protocol $\times$ set differences; *F*_(9, 171)_ = 86.9, *p* < 0.001) was observed between experimental protocols for HR response. In particular, CLU1 and CLU2 induced greater HR compared to TRA (CLU1 vs TRA, all *p* $<$ 0.001; CLU2 vs TRA, all *p* $<$ 0.001) and CLU4 (CLU1 vs CLU4, all *p* $<$ 0.001; CLU2 vs CLU4, all *p* $<$ 0.001) from the completion of the first sets throughout the entire sessions (Table 1 and Fig. 3).

**Fig. 4:**
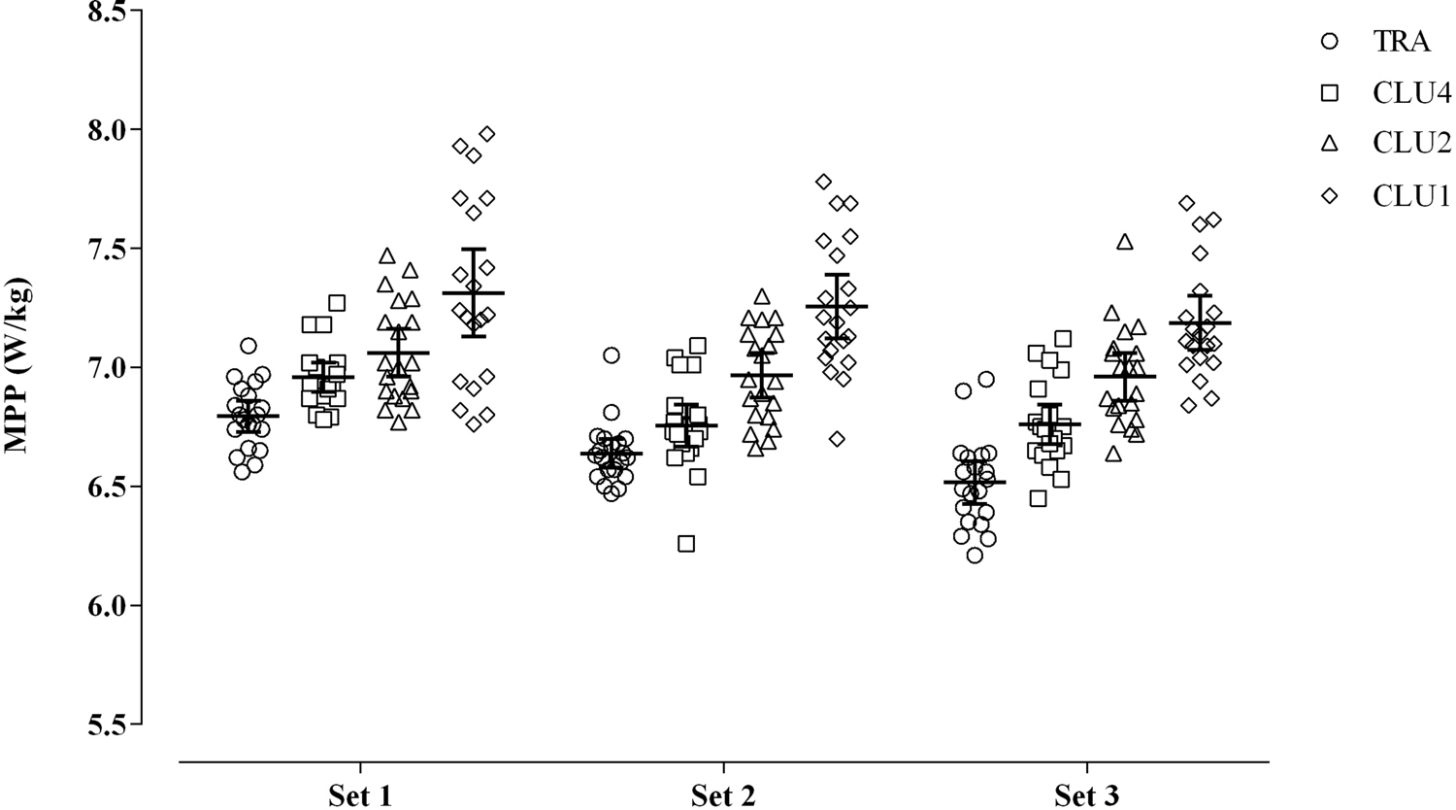
Comparison of the power outputs between the four different set configurations across sets. *MPP* mean propulsive power, *TRA* traditional-set configuration, *CLU1* single-repetition cluster configuration, *CLU2* double-repetition cluster configuration, *CLU4* quadruple-repetition cluster configuration

A main effect for protocol (*F*_(3, 57)_ = 62.5, *p* < 0.001) and protocol $\times$ set interaction (*F*_(6, 114)_ = 3.7, *p* = 0.002) was observed for MPP (Table 1; Fig. 4). Across all conditions, three consistent patterns emerged. First, cluster-set configurations consistently resulted in greater MPP compared to traditional-set configuration: CLU1 vs TRA (Set 1: 95% CI 0.42, 0.61; Set 2: 95% CI 0.52, 0.71; Set 3: 95% CI 0.57, 0.77), all *p* < 0.001; CLU2 vs TRA (Set 1: 95% CI 0.17, 0.36; Set 2: 95% CI 0.23, 0.42; Set 3: 95% CI 0.35, 0.54), all *p* < 0.001; CLU4 vs TRA (Set 1: 95% CI 0.07, 0.26; Set 2: 95% CI 0.02, 0.21; Set 3: 95% CI 0.15, 0.34), all *p* $\le$ 0.007. Second, a step-wise increase in MPP was observed when clusters went from high to low number of repetitions per cluster: CLU1 vs CLU4 (Set 1: 95% CI 0.26, 0.45; Set 2: 95% CI 0.4, 0.59; Set 3: 95% CI 0.33, 0.52), all *p* < 0.001; CLU1 vs CLU2 (Set 1: 95% CI 0.16, 0.35; Set 2: 95% CI 0.19, 0.38; Set 3: 95% CI 0.13, 0.32), all *p* < 0.001; CLU2 vs CLU4 (Set 1: 95% CI 0.01, 0.2; Set 2: 95% CI 0.11, 0.31; Set 3: 95% CI 0.1, 0.3), all *p* $\le$ 0.03 (Table 1; Fig. 4). Finally, a significant progressive decrease of power output was observed across the three consecutive sets during the TRA protocol (all *p* $\le$ 0.037). A similar pattern was also observed during CLU4 protocol, but differences reached significance only between set 1 and the two other sets (*p* < 0.001).

**Fig. 5:**
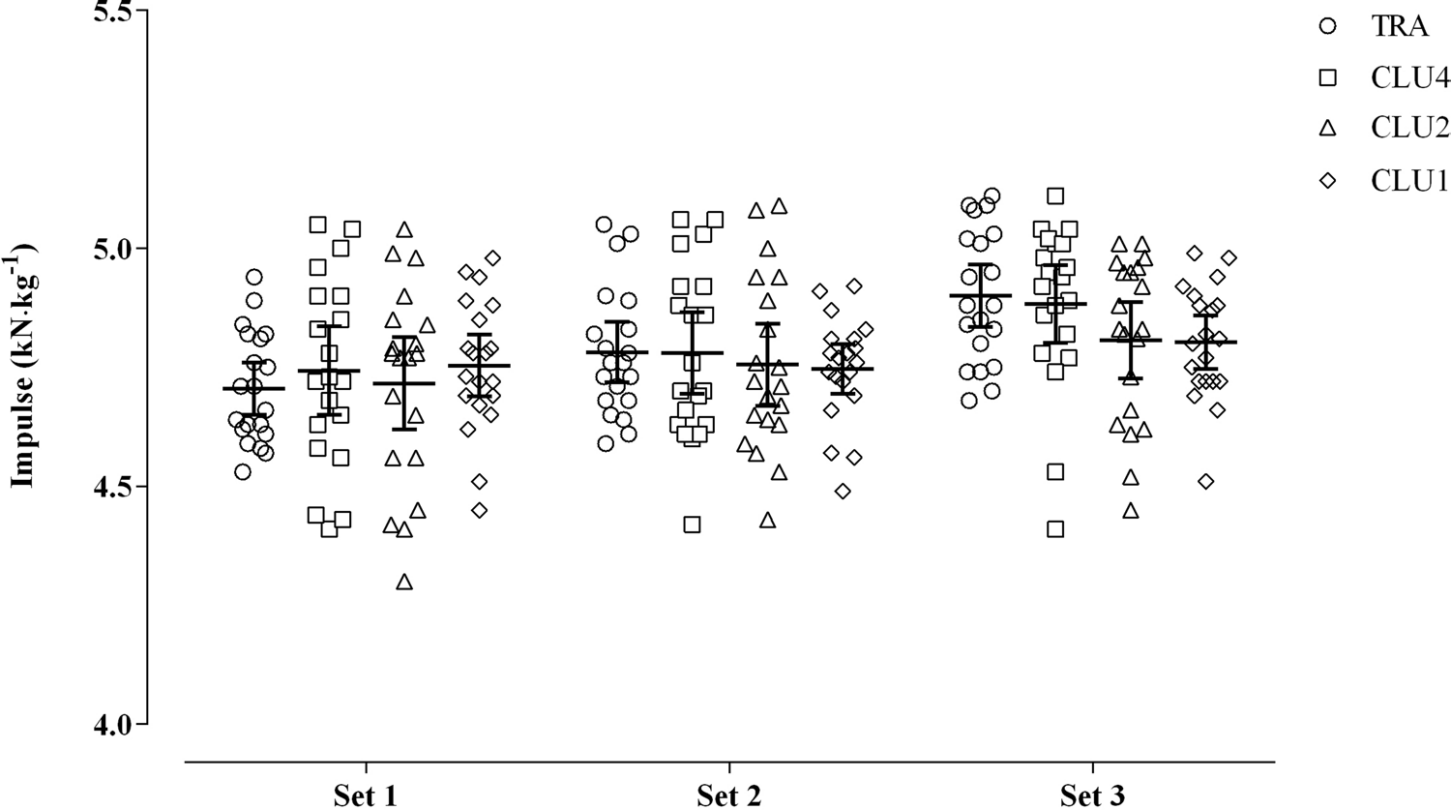
Comparison of the impulse outputs between the four different set configurations across sets. *TRA* traditional-set configuration, *CLU1* single-repetition cluster configuration, *CLU2* double-repetition cluster configuration, *CLU4* quadruple-repetition cluster configuration

No main effect (*F*_(3, 57)_ = 0.6, *p* = 0.62) was found between experimental protocols for impulse; however, a significant protocol $\times$ set interaction (*F*_(6, 114)_ = 4.9, *p* < 0.001) was observed (Table 1; Fig. 5). In particular, the traditional-set configuration and the cluster protocol with higher number of repetitions resulted in greater impulse outputs compared to the cluster protocols with low number of repetitions in set 3: TRA vs CLU1 (95% CI 0.04, 0.15, *p* < 0.001); TRA vs CLU2 (95% CI 0.04, 0.15, *p* < 0.001); CLU4 vs CLU1 (95% CI 0.03, 0.14, *p* < 0.001); CLU4 vs CLU2 (95% CI 0.02, 0.13, *p* = 0.002).

Significant differences were observed between the experimental protocols for RPE (*χ*^2^ = 57.4, *p* < 0.001) and ROF (*χ*^2^ = 50.5, *p* < 0.001), with a consistent pattern across protocols: CLU1 and CLU2 induced lower RPE [CLU1 vs TRA (95% CI − 3.2, − 1.8; *p* = 0.006); CLU2 vs TRA (95% CI − 2.6, − 1.4; *p* = 0.004); CLU1 vs CLU4 (95% CI − 3.2, − 1.7; *p* = 0.005); CLU2 vs CLU4 (95% CI − 2.7, − 1.3; *p* = 0.003)] combined with lower ROF scores [CLU1 vs TRA (95% CI − 4, − 2.2; *p* = 0.006); CLU2 vs TRA (95% CI − 3.6, − 2.1; *p* = 0.005); CLU1 vs CLU4 (95% CI − 1.4, − 0.17; *p* = 0.012); CLU2 vs CLU4 (95% CI − 1, − 0.15; *p* = 0.006)] compared to the TRA and CLU4 configurations. Lower ROF responses were also observed following CLU4 compared to TRA (95% CI − 1.5, − 3; *p* = 0.004). (Table 1). Finally, an absolute preference for the cluster-set conditions was observed with 15, 3 and 2 subjects choosing the CLU2, CLU1 and CLU4, respectively.

**Fig. 6:**
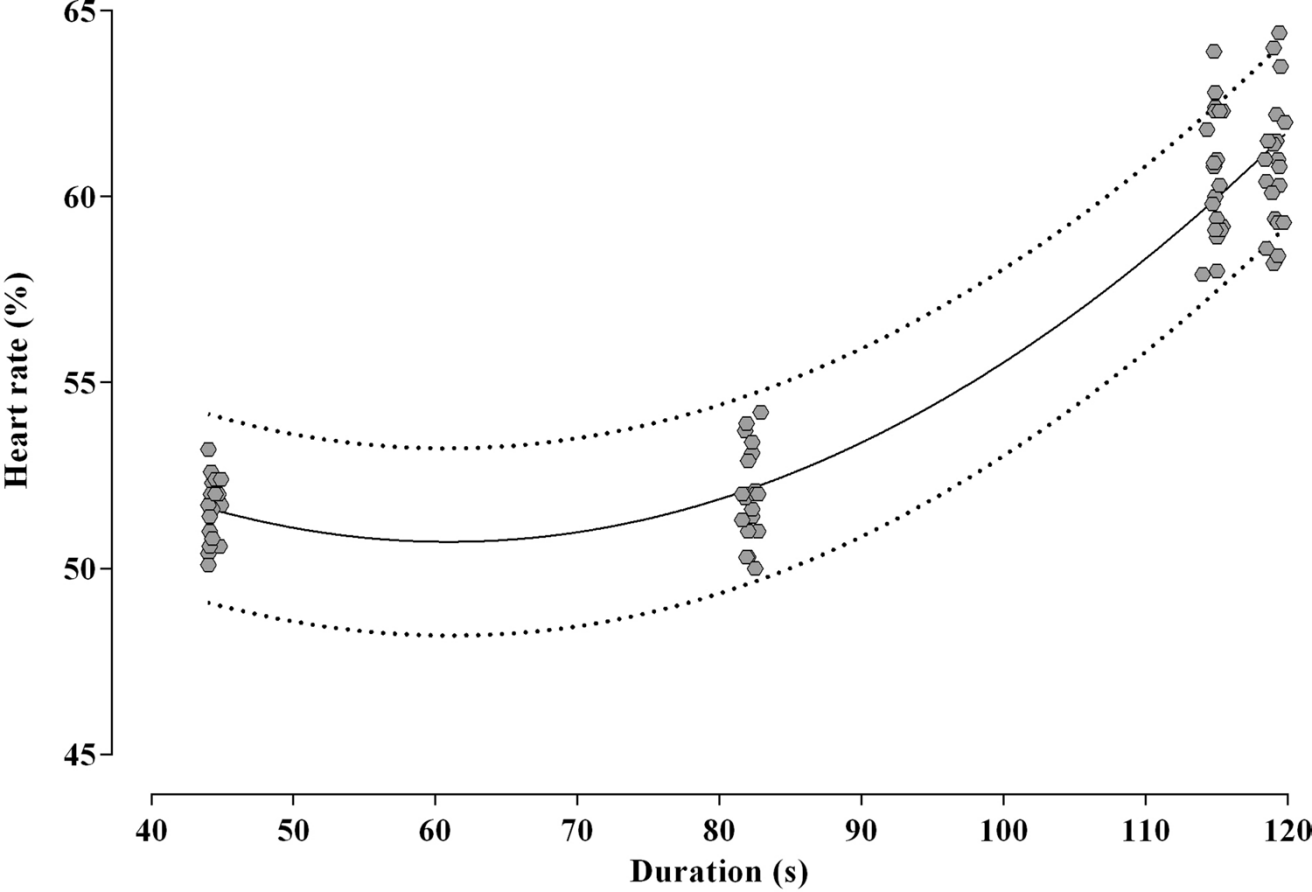
Scatterplot of the individual HR (%) responses as function of duration (s) of set 1 across protocols. The solid mid-line and dashed error lines represent the mean and 95% CI of the predicting fit

The relationship between HR response and duration of set 1 is shown in Fig. 6. The polynomial regression model confirmed HR was dependent on duration, which explained the variance HR with 90% confidence (i.e. *R*^2^ adjusted = 0.90; *p* < 0.001). The best-fit equation of the model is reported below:

$$HR=50.73+ 0.01483\mathrm{duration}+ 0.003162{\mathrm{duration}}^{2}$$

In practical terms, when the duration increases of 1 unit (i.e. 1 s) the HR is expected to change by 0.02%. For example, the model predicted an average difference in HR at completion of set 1 between CLU4 and CLU1 protocols of 9.1% (95% CI 8.7, 9.5) while the actual difference measured from data collected during the study was 8.7% (95% CI 7.7, 9.6).

## Discussion

In the current study, we examined the mechanical, physiological and perceptual responses to resistance training protocols designed with either traditional or cluster-set configurations in older men. Four main findings emerged: (1) cluster-set configurations resulted in greater MPP than traditional-set configuration; (2) clusters of lower repetition number (in this instance one and two) produced greater MPP than clusters of greater numbers (in this instance four); (3) clusters of lower repetition number induced greater HR and lower RPE and fatigue responses than traditional-set and clusters of greater numbers; and (4) cluster-set protocols were preferred over the traditional-set configuration, with clusters of two repetitions reported as the favorite.

Regardless of the set configuration, the three clusters protocols investigated in this study were associated with greater power outputs than the traditional-set protocol throughout the entire session. These findings are in agreement with previous investigations endorsing cluster-set configurations to optimize power outputs during the back squat exercise (Oliver et al. 2015, 2016b; Tufano et al. 2016; Wetmore et al. 2019). For example, in the study of Oliver et al. (2015) subjects performed back squats loaded with 70% of 1RM either as 4 sets of 10 repetitions with 120 s rest between sets, or as 4 sets of 2 clusters of 5 repetitions each with 30 s between clusters and 90 s between sets. Although the two training configurations were matched for exercise intensity, total volume and work: rest ratio, greater power outputs were observed during the cluster-set protocol. The authors concluded that the superior effects of the cluster-set configuration likely arose from the different rest structure and the inclusion of a short rest between consecutive clusters. Building on the findings of Oliver et al., we further examined the effects of cluster protocols designed with different configurations (one, two and four repetitions) and rest intervals duration (10, 20 and 40 s). The novel finding emerging from this study is that smaller clusters (one and two repetitions) including shorter (10–20 s) but more frequent rest intervals are an effective strategy to attenuate the velocity loss across repetitions and sets and to maximize power outputs. We assume that the rest interval embedded between clusters can reduce the rate of adenosine triphosphate (ATP) depletion and favor partial or complete regeneration of phosphocreatine (PCr) within the working muscles, thereby allowing maintenance of greater power outputs (Bogdanis et al. 1998; Gorostiaga et al. 2010, 2014). Moreover, we presume this mechanism may be exploited by implementing clusters with lower number of repetitions and frequent shorter rest intervals. Our assumption is supported by two main observations. The first, subjects produced greater power output during protocols with smaller clusters as confirmed by the stepwise trend of power output observed in this study (Fig. 3). Second, while power output decrement was present in all protocols, the decline was significant only during TRA and CLU4 set configurations (Table 1). In particular, power output decrements observed across consecutive sets during the TRA protocol, may have been caused by an acute increase in metabolic stress. This hypothesis is in agreement with previous studies, in which traditional-set configurations similar to the one used in this study led to greater blood lactate concentration and the consequent inability to maintain optimal power outputs (Girman et al. 2014; González-Hernández et al. 2020; Gorostiaga et al. 2014, 2010). As a result, prescribing resistance training using cluster-set configurations with low repetition numbers may help to avoid these detrimental metabolic effects and provide a greater stimulus for power enhancement adaptations than resistance training using traditional-set configuration and clusters of many repetitions. This is particularly pertinent in an aging population, as generating force at a high velocity is imperative to maintain mobility, independence and quality of life (Clark and Manini 2012; Runge et al. 2004). This hypothesis conforms to the findings of the study of Ramirez-Campillo et al. (2018) who reported greater improvements in functional performance and quality of life in older women (> 65 years) following a 12-week cluster-set configured resistance training programme compared to a matched programme with a traditional-set configuration and a control group.

The superior effects of cluster-set configurations with a low number of repetitions on power outputs are further supported by the HR data, whereby we observed greater HR responses during CLU1 and CLU2, than CLU4 and TRA across sets and throughout the entire session. These findings are in agreement with previous evidence suggesting the recovery within and between sets in resistance training sessions has an impact on cardiovascular responses (Fleck 1988, 2003; Kraemer et al. 1987). Differences in HR between protocols were already found at the completion of set 1 (Table 1; Fig. 4), most likely due to the significant longer duration of the same set in CLU1 ($\sim 118$ s) and CLU2 ($\sim 109$ s) compared to TRA ($\sim 45$ s) CLU4 ($\sim 82$ s), as confirmed by the results of the regression analysis between HR responses and set duration (Fig. 6). This suggests a greater metabolic demand during CLU1 and CLU2, and a greater aerobic training intensity and load, which could induce superior aerobic adaptation than the other protocols. In particular, the average HR responses achieved in CLU1 and CLU2 sessions were approximately 65% of HR_max_, thus within the 60–90% range that is recommended for the development of cardiorespiratory fitness and promotion of body composition changes (Medicine 2009; Pescatello et al. 2014). These findings have practical importance as resistance training with multiple exercises in cluster-set configurations could lead to superior cumulative peripheral and central stimuli and increase effective time within optimum training zones with consequent greater adaptations on both muscular and cardiovascular function.

As RPE and ROF were also lower when participants completed CLU1 and CLU2, it appears that these protocols, although exhibiting greater internal and external intensity, and subsequent load, were perceived as easier and less fatiguing. While the speculation mentioned previously concerning the likely differences between protocols in lactate concentration may hold true for greater perceived effort and fatigue following TRA and CLU4 protocols, we consider another reason to explain why such differences occurred. Greater impulses were observed in set 3 during TRA and CLU4 compared to CLU1 and CLU2 coupled with a dissimilar pattern for power outputs (i.e. CLU1 and CLU2 greater than TRA and CLU4 in set 3). The combination of greater impulse (i.e. force $\times$ time) and lower power (i.e. force $\times$ velocity [distance/time]) predicates longer duration for the eight repetitions in the last set, given that distance covered was constant. The longer duration of these repetitions in TRA and CLU4 may have caused greater perceptions of effort, fatigue and discomfort (Fisher and Steele 2017). Individuals tend to remember the peak and the end of an event (i.e. peak-end rule) (Hargreaves and Stych 2013; Kahneman et al. 1993), which in this study was the last set completed, and report perceptions accordingly. This also explains the divergent pattern which emerged between HR and RPE, although they are generally well correlated in response to exercise (Kraemer et al. 1987). In light of the above and in consideration that perception of effort and fatigue are key determinants of exercise adherence (Hartman et al. 2019; Prasad and Cerny 2002; Zenko et al. 2016), it seems prudent that CLU1 and CLU2 should be advocated when prescribing muscle strengthening exercises in older men. Finally, participants reported a preference for CLU2 over the other protocols (15, 3 and 2 for CLU2, CLU1 and CLU4, respectively). Taken together, results presented here suggest CLU2 may be the optimal resistance training design in older men, due to preference, low perception of effort and fatigue, greater HR responses and kinetic responses. CLU1 was superior in terms of MPP, and matched CLU2 in terms of HR and perception of effort and fatigue, yet it was not preferred by the majority of participants. Therefore, we feel it pragmatic to suggest CLU2 would be better received in this cohort.

This study is not without limitations. First, our participants were not resistance exercise naïve, being accustomed to this form of training. As exercise is a primary countermeasure to human aging, and the age-associated decline in muscle strength and mass, these individuals are not an at-risk cohort for frailty, so whether these findings translate to untrained individuals required further research. In this context, as our participants were trained, they presumably had a predilection for exercise, so perceptual responses may be different in this cohort compared to an exercise-adverse cohort. Second, the effects of the four protocols were investigated only on the back squat exercise loaded with OPL. This fact narrows the ability to generalize the results from this study to other conventional lower and upper body resistance training exercises across a broader range of loads and intensities. Finally, another limitation was the absence of additional physiological measurements (e.g., hormonal and lactate concentrations), which may have helped in better understanding the underlying mechanisms of resistance training under traditional and cluster-set configurations.

## Conclusions

The findings of the current study have few important practical implications. First, they suggest cluster-set configurations effectively maintain power outputs during the back squat, one of the most commonly prescribed lower limbs resistance training exercises (Tufano et al. 2017). Second, they indicate cluster-set configurations combining lower repetition numbers with shorter and more frequent rests lead to greater internal training load and low perception of effort and fatigue. Finally, cluster-set configurations are consistently preferred over a traditional-set configuration by trained older men. Consequently, it would be of great interest to investigate the long-term effects of different cluster-set modes on muscular and cardiovascular adaptations and functional performance of aging cohorts (Ramirez-Campillo et al. 2018). Future studies are warranted to elucidate the optimal combination of investigated protocols into a mixed approach, including aerobic exercise and balance training for older adults (Fragala et al. 2019).

## Abbreviations

1 RM: One-repetition maximum
ANOVA: Analysis of variance
ATP: Adenosine triphosphate
AU: Arbitrary unit
CLU: Cluster-set configuration
ES: Effect size
HR: Heart rate
HR_max_: Maximal heart rate
ICC: Intra-class Correlation Coefficient
MPP: Mean propulsive power
OPL: Optimum power load
PCr: Phosphocreatine
ROF: Rate of fatigue
RPE: Rate of perceived effort
SD: Standard deviation
TRA: Traditional-set configuration
$\beta$: Regression equation coefficient
$\varepsilon$: Regression equation random error
